# Neonatal pigs are susceptible to experimental Zika virus infection

**DOI:** 10.1038/emi.2016.133

**Published:** 2017-02-15

**Authors:** Joseph Darbellay, Kenneth Lai, Shawn Babiuk, Yohannes Berhane, Aruna Ambagala, Colette Wheler, Donald Wilson, Stewart Walker, Andrew Potter, Matthew Gilmour, David Safronetz, Volker Gerdts, Uladzimir Karniychuk

**Affiliations:** 1 Vaccine and Infectious Disease Organization-International Vaccine Centre (VIDO-InterVac), University of Saskatchewan, Saskatoon, SK, S7N 5E3, Canada; 2 Department of Veterinary Microbiology, University of Saskatchewan, Saskatoon, SK, S7N 5B4, Canada; 3 Canadian Food Inspection Agency, National Centre for Foreign Animal Disease, 1015 Arlington Street, Winnipeg, MB, R3E 3M4, Canada; 4 Canada National Microbiology Laboratory, Public Health Agency of Canada, 1015 Arlington Street, Winnipeg, MB, R3E 3R2, Canada

**Dear Editor,**

The current Zika virus (ZIKV; genus *Flavivirus*) epidemic in the Western Hemisphere has been declared a public health emergency. The virus can cause transplacental infections in pregnant women, resulting in microcephaly of the fetus, intrauterine growth restriction and abortions.^1^ In addition, regions with the heaviest ZIKV burden are endemic for dengue virus (DENV). It is possible that ZIKV infection or vaccination may enhance DENV infections, and render mild dengue fever into a life-threatening disease.^2^ Animal models to study ZIKV infection and ZIKV co-infections with other flaviviruses are crucial for further fundamental studies and development of effective interventions. To date, significant efforts have been made to develop only mouse and non-human primate models^3^ for ZIKV infection. The identification of another mammalian species able to at least partially model ZIKV infections in humans has significant value. It has been previously reported that flaviviruses, including DENV,^4^ West Nile virus^5^ and Japanese encephalitis virus,^6^ can infect domestic pigs. Therefore, we designed this preliminary study to determine whether neonatal pigs are susceptible to ZIKV infection.

First, we determined the susceptibility of one-day-old piglets to ZIKV infection. Two pregnant Landrace-cross sows were housed at the VIDO-InterVac. After farrowing, newborn piglets were challenged with 1  mL of inoculum containing 5.8 log_10_ 50% tissue culture infectious dose (TCID_50_)/mL of ZIKV strain PRVABC59 (GenBank: KU501215), which was isolated from human serum collected in Puerto Rico in December 2015 and was provided by the Centers for Disease Control and Prevention, Division of Vector-Borne Diseases, Fort Collins, Colorado, USA. Virus stocks were prepared by inoculating a confluent monolayer of Vero E6 cells. We selected a viral challenge dose within the previously reported range for West Nile virus: 10^2^–10^7^ infectious viral particles during a mosquito bite.^7^ A prior study also indicated a ZIKV load of up to 10^8^ TCID_50_ in the salivary gland of the *Aedes aegypti* mosquito.^8^ Eleven piglets were inoculated intracerebrally (IC), six piglets were inoculated intradermally (ID) in four sites on the neck skin and six piglets were inoculated intraperitoneally (IP). Three control mock-inoculated (IC-inoculated) and non-manipulated piglets were also included in the study. Animals were monitored daily for clinical signs. Blood was collected at three, five and seven days post virus inoculation (dpi). At each sampling time point, two piglets in the ID and IP groups and three to four piglets in the IC group were euthanized to collect urine (aspirated from the bladder), brain and spleen. Tissues were snap-frozen for virological analyses. A previously published ZIKV-specific real-time reverse transcription polymerase chain reaction (RT-PCR) SYBR Green assay was used to identify and quantify ZIKV RNA. Zika virus titers were identified by inoculation on Vero E6 cells and subsequent staining with ZIKV-specific antibodies (Ab; anti-ZIKV rabbit polyclonal Ab; IBT BIOSERVICES, MD, USA). The infectious virus in brain tissues was also isolated on mosquito C6/36 cells. For quantification of ZIKV-specific IgM, IgG Ab and neutralizing Ab (NAb), modified immunoperoxidase monolayer assay and neutralizing assay were used, respectively. A detailed description of experimental methods is provided in Supplementary Methods.

Mock-inoculated and non-manipulated animals did not exhibit any clinical signs, ZIKV RNA, live virus titers or virus-specific Ab. At 5 dpi, two ZIKV IC-inoculated piglets of eleven exhibited leg weakness, ataxia and tremor (Supplementary Videos 1 and 2). Similar clinical signs have been reported in ZIKV-infected neonatal mice^9^ and a human adult who died because of encephalitis associated with ZIKV infection.^10^ In the IC-inoculated piglets, ZIKV RNA in sera was detected at 3, 5 and 7 dpi, whereas ID- and IP-inoculated piglets were positive at 3 and 5 dpi (Figure 1A). In the IC-inoculated piglets, the live virus was detected in sera at 3 and 5 dpi (Figure 1B). Intradermally and IP-inoculated piglets had live ZIKV at 3 dpi, except one ID-inoculated piglet that was positive at 5 dpi. Analysis of variance on ranks exhibited significant differences between IC and IP groups at 5 dpi (*P*=0.02). ZIKV RNA and live virus were also identified in urine (IC and ID groups), brain (only IC group) and spleen (IC, ID and IP groups; Supplementary Table S1). Brain samples were negative for infectious ZIKV on Vero E6 cells. From personal communications with other researchers and publications on the conventional mouse^11^ and non-human primate models,^12^ we know that Vero E6 cells may be not sensitive enough for ZIKV isolation from tissues. Alternatively, a load of infectious virus in brain tissues from conventional animals may be too small to be isolated on Vero E6 cells. However, inoculation of more sensitive C6/36 cells with brain suspensions (Supplementary Materials) resulted in the isolation of infectious ZIKV (Supplementary Table S1). Serum samples from all groups were positive for ZIKV IgM Abs (IC group - 2–4 log_2_; ID and IP groups - 3 log_2_) at 7 dpi. In addition, weak NAb activity was observed in sera (IC group – 2–2.6 log_2_; ID – 1.6-3.6 log_2_; and IP – 1.6–2 log_2_) at 7 dpi.

Second, to further confirm the infectivity of ZIKV in tissue and serum samples from challenged animals and the susceptibility of pigs to the lower infectious doses, we performed a serial passage experiment. In addition, we verified the feasibility of experimental ZIKV passage on pigs given that human viruses adapted to animals via serial passage might be a useful tool to study disease pathogenesis, virus interactions with a new host and viral evolution. In the serial passage experiment, two newborn piglets (Figure 1C – Passage 1) were inoculated IC with 1 mL of serum or 1 mL of brain suspension in PBS (1:1 volumes) obtained from a piglet inoculated IC with 1 mL of inoculum containing 5.8 log_10_ TCID_50_ of ZIKV (Figure 1C – Initial challenge). Both newborn piglets inoculated IC with either the pig serum or brain suspension containing low ZIKV doses (serum=1.8 log_10_ TCID_50_/mL; brain suspension <1.2 log_10_ TCID_50_/g) had viremia at 4–6 dpi (Figure 1C – Passage 1). We used sera collected at 6 dpi from the piglet inoculated with brain suspension to perform the second IC passage in two older, six-day-old piglets. These animals developed viremia at 4–6 dpi (Figure 1C – Passage 2) and high titers of ZIKV IgM and IgG Abs at 9–14 and 10–14 dpi, respectively (Figure 1D). IgM and IgG Abs were primarily detected two days later than in the first challenge experiment, and this effect was likely attributable to the much lower ZIKV inoculation dose. Piglets also developed moderate NAb responses at 9–14 dpi (Figure 1D). Kinetics of ZIKV Ab responses in pigs were similar to those in humans.^13^ Neutralizing Ab titers in pigs were also similar to NAb responses in experimentally inoculated rhesus macaques.^3^

In this preliminary study, we found that ZIKV infects conventional neonatal pigs, causing viremia, viruria and virus replication in internal organs. We also demonstrated that one-day-old and six-day-old piglets are susceptible to a low infectious dose of ZIKV and that experimental serial passage is feasible. The serial passage of ZIKV on pigs may be a useful approach to generate ZIKV strains for pathogeneses and evolutionary studies.

Most importantly, pigs inoculated with ZIKV via different routes developed IgM, IgG, and neutralizing Abs, which may provide opportunities to study the antibody-dependent enhancement (ADE) of other flavivirus infections. For example, the major risk factor for developing severe dengue fever is ADE of infection caused by the presence of DENV-reactive Ab from a previous infection with a different DENV serotype. Antibodies induced by previous infections with other related flaviviruses may cause the same detrimental effects. In support, polyclonal mouse Ab against ZIKV enhanced DENV infection *in vitro.*^2^ Transfer of ZIKV monoclonal Ab to immunocompromised mice also enhanced subsequent DENV infections.^14^ Thus, ZIKV vaccination or infection may enhance DENV infections and render mild dengue fever into a life-threatening disease. To the authors' knowledge, ZIKV co-infections with DENV or other flaviviruses have not been tested *in vivo*. Previously published mouse models of ADE during DENV infection utilize immunocompromised mice and passive Ab transfer. The work on non-human primate is not feasible to most research groups and is constrained by economic boundaries. As an alternative, conventional pigs are susceptible to DENV^4^ and to other human flaviviruses, including West Nile virus^5^ and Japanese encephalitis virus.^6^ Furthermore, human infants have the major risk of developing severe dengue fever as a result of ADE of infection due to DENV-reactive Ab acquired from an immune mother.^15^ Thus, neonatal pigs might be used to model ZIKV and DENV (or another flavivirus) co-infections and ADE of infection in infants.

Beyond modeling ZIKV infection, our data may have epidemiological applications. Given that ZIKV is an emerging pathogen in the Americas, it is difficult to predict potential reservoirs of the virus as it spreads in the new environment. Japanese encephalitis virus, another member of the family Flaviviridae, uses pigs as amplifying hosts with subsequent mosquito-mediated transmission to humans. Therefore, additional studies are necessary to clarify if ZIKV has the potential to establish endemic infections in swine herds and a sylvatic cycle involving pigs.

## Figures and Tables

**Figure 1:**
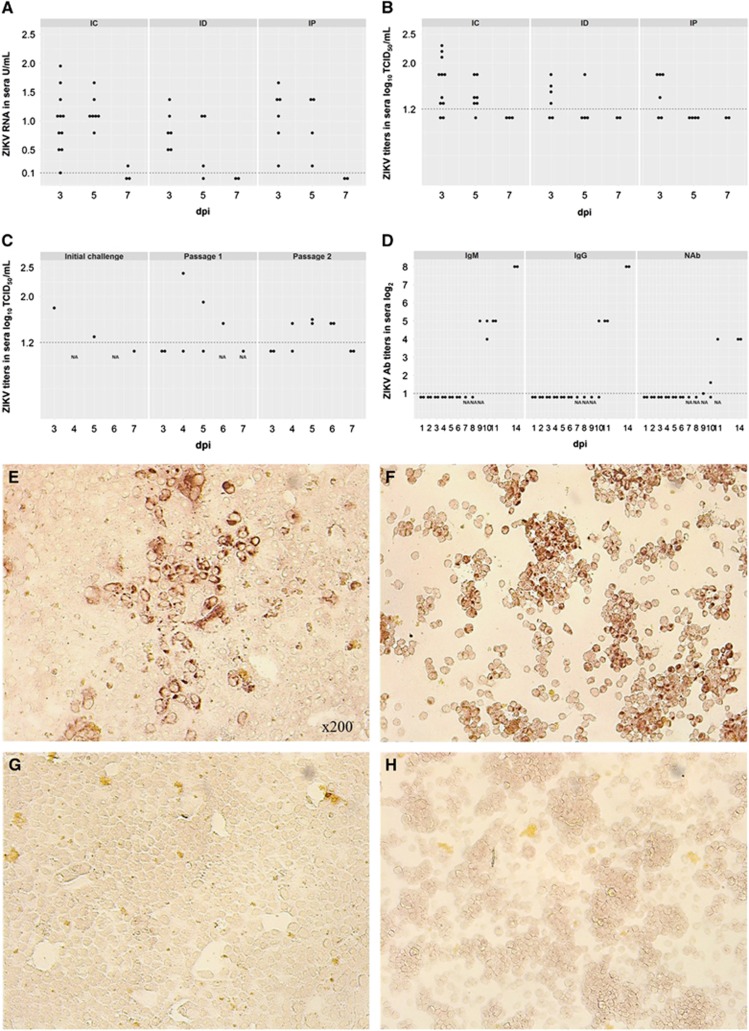
Dots represent results in each sample collected from each experimental animal used in the study. A dotted line represents the assay detection limit. Dots below the detection limit are negative samples. (**A**) Relative ZIKV PCR titers in sera of piglets inoculated IC, ID and IP. Relative viral loads were determined using RNA from a stock of ZIKV with a known TCID_50_ titer. Relative log_10_ TCID_50_ values were defined as RNA units (U) and expressed as ZIKV RNA U per mL. (**B**) Infectious ZIKV TCID_50_ titers in sera of piglets inoculated IC, ID and IP. (**C**) ZIKV serial passage in piglets. Initial challenge – A piglet (piglet #177 Supplementary Table S1) was inoculated IC with 1 mL of inoculum containing 5.8 log_10_ TCID_50_ of ZIKV. Subsequently, serum (collected at 3 dpi) and brain tissues (collected at 7 dpi) from this piglet were used for passage 1. Passage 1 – Two newborn piglets were inoculated IC with 1 mL serum or 1 mL brain suspension (serum=1.8 log_10_ TCID_50_/mL; brain<1.2 log_10_ TCID_50_/g) from the ZIKV-infected piglet. The piglet inoculated with serum developed viremia at 5 dpi. The piglet inoculated with brain suspension developed viremia at 4 and 6 dpi. Passage 2 – Serum from passage 1 collected at 6 dpi was used to inoculate two six-day-old piglets IC. (**D**) Antibody titers in pigs from Passage 2. Vero E6 (**E**) and C6/36 (**F**) cells inoculated with ZIKV-positive serum and brain tissues, respectively. Vero E6 (**G**) and C6/36 (**H**) cells inoculated with samples from control pigs. The infectious ZIKV was identified by immunohistochemistry as described in Supplementary Methods. Brown staining represents active viral replication in cells. Intracerebrally, IC; intradermally, ID; intraperitoneally, IP; neutralizing antibody, NAb.
