# Supplementary material for: Neonatal pigs are susceptible to experimental Zika virus infection
Source: Emerg Microbes Infect. 2017 Feb 15;6(2):e6–. doi: 10.1038/emi.2016.133 (PMC5322322; doi:10.1038/emi.2016.133)
Supplement: Supplementary Materials and methods [file emi2016133x1.docx]

**Supplementary Materials and Methods**

*Real-time RT-PCR*

Viral RNA from serum and urine samples was extracted using QIAamp Viral RNA Mini Kit (Qiagen). Tissues were weighed and homogenized into 600 μL RLT buffer (spleen) or 1ml QIAzol Lysis Reagent (brain) using RNase-free stainless steel beads and TissueLyser II (Qiagen) operating for 5 min at 25 Hz. RNA was extracted using the Qiagen RNeasy Mini extraction kit (spleen) or Qiagen RNeasy Lipid Tissue Mini Kit (brain). A previously published ZIKV specific real-time RT-PCR SYBR Green assay was used for Zika virus (ZIKV) RNA quantification^1^. As a positive PCR control we used Vero E6 cell culture media containing ZIKV. As a negative control we used samples from mock-inoculated and non-manipulated piglets. Strict precautions were taken to prevent PCR contamination. Aerosol-resistant pipette tips and disposable gloves were always used. Reagent controls, with water instead of body fluid/tissue samples were included in every RNA isolation and PCR run. All PCR reactions were conducted with SensiFAST Probe Hi-ROX One-Step Kit and SensiFAST SYBR & Fluorescein One-Step Kit reagents (Bioline) on the StepOne Plus platform (Life Technologies) and analysed using StepOne software version 2.3. Relative viral loads were determined using RNA from a stock of ZIKV with a known TCID_50_ titer to generate a PCR standard curve. For convenience, relative log_10_ TCID_50_ values were defined as RNA units (U), and expressed as ZIKV RNA U per ml of serum or urine. Relative values from tissues samples were corrected for their weight and expressed as ZIKV RNA U per mg.

*Virus titration on Vero E6 cells*

Tissue homogenates and body fluids were serially diluted twofold in four replicates starting at a dilution of 1:2, and 50μl of each dilution was added to confluent Vero E6 cells cultured in 96-well plates in DMEM (Sigma) supplemented with 10% fetal bovine serum (FBS) (Sigma). After 2h of incubation, the inoculum was removed, cells were washed and fresh media (2% FBS) was added. The cells were incubated for 5 days before plate washing and drying. The plates were kept at -20°C until use. Plates were thawed and then fixed in 10% buffered formalin (Fisher Chemical) for 15 min, washed twice with PBS and incubated with 0.33% Triton X-100 (Fisher Bioreagents) and 1% of 30%H_2_O_2_ (Sigma) for 20 min. Plates were washed twice with PBS and 50μl of affinity-purified rabbit anti-ZIKV polyclonal antibodies (Ab) (1.3µg/ml working dilution; IBT BIOSERVICES; peptide sequence to ZIKV E glycoprotein was used as immunogen to produce Ab; Ab are verified in Western blot and ELISA by a manufacturer) with 10% goat serum (Gibco) were added per well, followed by incubation at 37°C for 1h. Plates were washed three times with PBS containing 0.05% Tween 80 and 50μl of goat anti-rabbit IgG (1/2000 working dilution; Abcam) conjugated with horseradish peroxidase (HRP) with 10% goat serum were added per well, followed by incubation at 37°C for 1h. Plates were washed three times and 50μl of Lab Vision™ Ready-To-Use AEC Substrate System (Thermo Scientific™) were added to each well, after which plates were incubated at room temperature for 20 min. Then, the reaction was halted by replacing the substrate with an acetate buffer, and ZIKV-specific staining was determined by examination with a microscope. ZIKV titers were determined using the Reed and Muench method^2^. As a positive control we used Vero E6 cell culture media containing ZIKV. Body fluid and tissue samples from mock-inoculated and non-manipulated piglets were used as negative controls.

*Virus isolation on C6/36 cells*

Sensitive cell culture-based procedure for isolating the infectious ZIKV on C6/36 cells was previously described^3^. The brain homogenates positive for ZIKV by PCR were inoculated directly into 96-well plates containing C6/36 cells. The infected cells were cultured at 28°C in 5% CO_2_ for 7 days. On day 7, the culture supernatant was collected and passaged on naïve C6/36 cells. Serial passages were conducted two times. The viral RNA was extracted from culture supernatant at each passage, and RT-PCR was performed as described above. To confirm the isolation of the virus, ZIKV-specific immunohistochemistry assay on fixed C6/36 cells was performed as described above. The increase in PCR Ct values and specific staining indicated the presence of infectious ZIKV. As a positive control we used cell culture media containing ZIKV. Brain homogenates from mock-inoculated and non-manipulated piglets were used as negative controls.

*Antibody responses*

For quantification of ZIKV-specific IgM, IgG Ab and neutralizing Ab (NAb) modified immunoperoxidase monolayer assay (IPMA)^4^ and neutralizing assay^5^ were used, respectively. Vero E6 cells in 96-well cell culture plates were inoculated with 50μl media containing 2.3 log_10_ TCID_50_/ml ZIKV and incubated for 72h (37°C, 5% CO_2_). Then, the culture media was removed and plates were washed and dried. The plates were kept at -20°C until use. Plates were thawed and then fixed in 10% buffered formalin for 15 min. Cells were washed twice with PBS and incubated with 0.33% Triton X-100 and 1% of 30% H_2_O_2_ for 20 min. Plates were washed twice with PBS and two-fold serial dilutions of heat-inactivated sera (56°C for 30 min) were added, followed by incubation for 1 h at 37°C. Plates were washed three times with PBS containing 0.05% Tween 80 and after addition of 50μl of goat anti-swine IgM (1/20 working dilution; KPL) or rabbit anti-pig (1/100 working dilution; Abcam) Abs conjugated with HRP per well, plates were incubated at 37°C for 1h. Afterward, plates were washed and Lab Vision™ Ready-To-Use AEC Substrate System (Thermo Scientific™) was added. Specific staining was determined as described above. Sera from mock-inoculated and non-manipulated piglets were used as negative controls.

To determine titers of NAb, 50µl of ZIKV (2 log_10_ TCID_50_/ml) were mixed with equal volumes of heat-inactivated, serially 1:2 diluted sera (in two replicates) and incubated at 37°C for 1 hour, before inoculation onto Vero E6 cells in 96-well plates. After 72h, cells were fixed and stained with ZIKV-specific Ab as described for virus titration. The NAb titer was defined as the reciprocal of the highest serum dilution that inhibited ZIKV infection in 50% of the inoculated wells. Sera from mock-inoculated and non-manipulated piglets were used as negative controls.

**References**

1 Xu M-Y, Liu S-Q, Deng C-L, Zhang Q-Y, Zhang B. Detection of Zika virus by SYBR green one-step real-time RT-PCR. *J Virol Methods* 2016; **236**: 93–97.

2 Reed LJ, Muench H. A simple method of estimating fifty per cent endpoints,. *Am J Epidemiol* 1938; **27**: 493–497.

3 Deng C, Liu S, Zhang Q, Xu M, Zhang H, Gu D *et al.* Isolation and characterization of Zika virus imported to China using C6/36 mosquito cells. *Virol Sin* 2016; **31**: 176–9.

4 Wensvoort G, Terpstra C, Pol JMA, ter Laak EA, Bloemraad M, de Kluyver EP *et al.* Mystery swine disease in the Netherlands: The isolation of Lelystad virus. *Vet Q* 1991; **13**: 121–130.

5 Lefebvre DJ, Costers S, Van Doorsselaere J, Misinzo G, Delputte PL, Nauwynck HJ. Antigenic differences among porcine circovirus type 2 strains, as demonstrated by the use of monoclonal antibodies. *J Gen Virol* 2008; **89**: 177–187.
