# Supplementary material for: Neonatal pigs are susceptible to experimental Zika virus infection
Source: Emerg Microbes Infect. 2017 Feb 15;6(2):e6–. doi: 10.1038/emi.2016.133 (PMC5322322; doi:10.1038/emi.2016.133)
Supplement: Supplementary Table S1 [file emi2016133x2.docx]

**Supplementary Table S1** Zika virus (ZIKV) in urine and tissue samples

| Pig  No | Inoculation | Urine* | | |  |  | Brain^ǂ^ |  |  |  | Spleen^ǂ^ |  |
| --- | --- | --- | --- | --- | --- | --- | --- | --- | --- | --- | --- | --- |
|  |  | 3 dpi | 5 dpi | 7 dpi |  | 3dpi | 5 dpi | 7dpi |  | 3 dpi | 5 dpi | 7 dpi |
| 167 | IC | -/- |  |  |  | 0.7/- |  |  |  | -/- |  |  |
| 170 | IC | -/- |  |  |  | 0.1/- |  |  |  | -/- |  |  |
| 159 | IC | -/- |  |  |  | 0.9/- |  |  |  | -/- |  |  |
| 162 | IC | -/- |  |  |  | 0.8/- |  |  |  | 1.1/- |  |  |
| 171 | IC |  | 0.1/**2.0** |  |  |  | 0.7/- |  |  |  | -/- |  |
| 174 | IC |  | - |  |  |  | 0.8/- |  |  |  | -/- |  |
| 156 | IC |  | 0.2/**1.5** |  |  |  | -/- |  |  |  | 0.2/**2.4** |  |
| 164 | IC |  | 0.2/**1.5** |  |  |  | 0.2/- |  |  |  | 0.2/**2.8** |  |
| 177 | IC |  |  | -/- |  |  |  | 1.4/- |  |  |  | -/- |
| 178 | IC |  |  | -/- |  |  |  | -/- |  |  |  | -/- |
| 163 | IC |  |  | 0.1/**1.6** |  |  |  | 0.8/- |  |  |  | -/- |
|  |  |  |  |  |  |  |  |  |  |  |  |  |
| 169 | ID | -/- |  |  |  | -/- |  |  |  | 0.8/- |  |  |
| 161 | ID | 0.1/- |  |  |  | -/- |  |  |  | 0.7/- |  |  |
| 173 | ID |  | -/- |  |  |  | -/- |  |  |  | -/- |  |
| 154 | ID |  | -/- |  |  |  | -/- |  |  |  | 1.0/- |  |
| 176 | ID |  |  | -/- |  |  |  | -/- |  |  |  | -/- |
| 158 | ID |  |  | -/- |  |  |  | -/- |  |  |  | 1.0/- |
|  |  |  |  |  |  |  |  |  |  |  |  |  |
| 168 | IP | -/- |  |  |  | -/- |  |  |  | 0.5/- |  |  |
| 153 | IP | -/- |  |  |  | -/- |  |  |  | -/- |  |  |
| 172 | IP |  | -/- |  |  |  | -/- |  |  |  | -/- |  |
| 160 | IP |  | -/- |  |  |  | -/- |  |  |  | 0.3/- |  |
| 175 | IP |  |  | -/- |  |  |  | -/- |  |  |  | -/- |
| 157 | IP |  |  | -/- |  |  |  | -/- |  |  |  | -/- |

*ZIKV RNA U per ml of urine/**ZIKV titers log_10_ TCID_50_ per ml of urine**.

^ǂ^ ZIKV RNA U per g of tissue/**ZIKV titers log_10_ TCID_50_ per g of tissue**.

ZIKV in tissues was titrated on Vero E6 cells as described in supplementary methods. In highlighted samples infectious ZIKV was isolated on mosquito C6/36 cells as described in supplementary methods.

“-”, values below the detection limit – 0.1 RNA U per ml of urine or g of tissue; 1.2 log_10_ TCID_50_ per ml of urine or g of tissue.

IC, intracerebral; ID, intradermal; IP, intraperitoneal.

dpi, days post-virus inoculation and day of euthanasia.
